# Rapid Maxillary Anterior Teeth Retraction *En Masse* by Bone Compression: A Canine Model

**DOI:** 10.1371/journal.pone.0026398

**Published:** 2011-10-19

**Authors:** Chufeng Liu, Yang Cao, Conghua Liu, Jincai Zhang, Pingping Xu

**Affiliations:** 1 Department of Orthodontics, Guangdong Provincial Stomatological Hospital, Southern Medical University, Guangzhou, China; 2 Department of Orthodontics, Guanghua College of Stomatology, Sun Yat-sen University, Guangzhou, China; 3 Department of Periodontology, Guangdong Provincial Stomatological Hospital, Southern Medical University, Guangzhou, China; 4 Department of Oral and Maxillofacial Surgery, Guangdong Provincial Stomatological Hospital, Southern Medical University, Guangzhou, China; Harvard Medical School, United States of America

## Abstract

**Objective:**

The present study sought to establish an animal model to study the feasibility and safety of rapid retraction of maxillary anterior teeth *en masse* aided by alveolar surgery in order to reduce orthodontic treatment time.

**Method:**

Extraction of the maxillary canine and alveolar surgery were performed on twelve adult beagle dogs. After that, the custom-made tooth-borne distraction devices were placed on beagles' teeth. Nine of the dogs were applied compression at 0.5 mm/d for 12 days continuously. The other three received no force as the control group. The animals were killed in 1, 14, and 28 days after the end of the application of compression.

**Results:**

The tissue responses were assessed by craniometric measurement as well as histological examination. Gross alterations were evident in the experimental group, characterized by anterior teeth crossbite. The average total movements of incisors within 12 days were 4.63±0.10 mm and the average anchorage losses were 1.25±0.12 mm. Considerable root resorption extending into the dentine could be observed 1 and 14 days after the compression. But after consolidation of 28 days, there were regenerated cementum on the dentine. There was no apparent change in the control group. No obvious tooth loosening, gingival necrosis, pulp degeneration, or other adverse complications appeared in any of the dogs.

**Conclusions:**

This is the first experimental study for testing the technique of rapid anterior teeth retraction *en masse* aided by modified alveolar surgery. Despite a preliminary animal model study, the current findings pave the way for the potential clinical application that can accelerate orthodontic tooth movement without many adverse complications.

**Clinical Relevance:**

It may become a novel method to shorten the clinical orthodontic treatment time in the future.

## Introduction

Distraction osteogenesis (DO) is manifested as rapid new bone growing by the mechanical stretching of the pre-existing bone tissue which takes advantage of osseous remodeling capabilities of the callus at the osteotomy and/or corticotomy sites. This innovative concept of bone biology opens a new vista for minimally invasive treatment of jaw deformities. So far, DO technique has achieved great success in the treatment of severe bone deficiencies, including micrognathia, sequelae of cleft lip and palate, and maxillofacial bone defects. In stark comparison, very few advances have been in using DO technique to treat excessive bone disorders such as prognathism. Based on the mechanical principle of DO, it will be fascinating to know the biological response when the bone is imposed on the compression force by reverse activating of the distractor, usually applied in DO for supplying the distraction force.

Maxillary protrusion is a common dentognathic deformity. Orthodontics and orthodontics combined with anterior segmental osteotomy are the common treatment strategies. The combined orthodontic and anterior segmental osteotomy therapy can markedly reduce the length of treatment over the conventional orthodontic treatment and result in immediate improvement of the facial profile. But its various postoperative complications including ischemic necrosis of the anterior segment, wound dehiscence at the osteotomy site, and devitalization of the teeth adjacent to the osteotomy site deter many patients from seeking the treatment [1]. The more conventional and commonly-used orthodontic treatment for maxillary protrusion heavily relies on the biological tooth movement [2], which happens at a limited rate and thus prolongs the treatment to 2 years for most patients [3]. Even more time is required for adult patients, who often wish their treatment could be completed as soon as possible [4].

In clinical studies, osteotomies or corticotomies, defined as the osteotomies of the cortical bone, have been combined with orthodontics to accelerate the tooth movement [5]. Among these procedures, alveolar corticotomies have been used for many years. Selective buccal and lingual decortication of the alveolar bone is commonly used to accelerate orthodontic tooth movement [6]. Several studies suggest that bone response with corticotomy occurs by regional accelerated phenomenon (RAP), which induces demineralization in the alveolar bone around the dental roots. RAP is initially derived from the rare cases of fracture healing [7], [8]. The term “regional” refers to the demineralization of both the cut site and the adjacent bone. The term “acceleratory” refers to an intensified bone response in cuts which extends to the marrow [9]. This technique dramatically reduces the treatment time because once the bone has demineralized, there is an opportunity to move teeth rapidly through the demineralized bone matrix before the alveolar bone remineralizes [5], [6], [10]–[13]. The alveolar corticotomy technique has been modified over the years to eliminate possible risks of the procedure, including periodontal damage, devitalization of the teeth and osseous segments because of inadequate blood supply.

Similar to DO, “distraction of periodontal ligament” was first conceptualized in 1998 [14] and later in 2002 another similar term “dentoalveolar distraction osteogenesis” was created [15]. The basic idea behind these concepts is to use a tooth-borne, custom-made intraoral distraction device to move the canines at a rate of 0.5 to 1.0 mm per day towards the distal end after the first premolar extraction. Their clinical applications prove to be successful: the duration of orthodontic treatment is greatly shortened by several months and no clinical and radiographic evidence of complications such as root fracture, root resorption, ankylosis, or periodontal problems is ever observed. However, these reports mainly focus on the movement of a single tooth and are confined in theoretical frame of DO.

Based on previous studies, we hypothesized that the maxillary bone could be compressed to achieve rapid maxillary anterior teeth retraction *en masse*. In this pilot study, a tooth-borne distraction device is introduced to six maxillary anterior teeth to investigate the feasibility and safety of rapid bulk adduction of these teeth aided by modified alveolar corticotomy technique. Since stronger force is needed to compress the bone than the single tooth, its influence on root resorption and periodontal tissue is the focus of this preliminary study.

## Results

### Gross alterations

All animals tolerated the operation and compressive procedures fine and their wounds healed well without infection. Additionally, the beagle dogs did not markedly lose weight. Though they initially lost less than 5% of total weight, about 0.5–0.6 kg, 2 weeks post-surgery, their weight recovered to preoperative levels 4 weeks post-surgery. At 6-week post-surgery, the weight of some animals even increased by 1.0 kg. The compressive devices remained in place and intact until they were removed.

At the end of compression, anterior crossbite was evident in the experimental group, whereas there was no obvious change in control group. No loosening of teeth, soft tissue dehiscence or bone necrosis in the operated region appeared. The total amounts of incisor and premolar movements after consolidation for 1, 14 and 28 days are shown in Table 1. The average total movements of incisors within 12 days were 4.63±0.10 mm and anchorage losses were 1.25±0.12 mm. After 14 to 28 days of consolidation, the average movements of incisors decreased slightly, but no significant difference was found among the variables in after 1, 14 and 28 days of consolidation. Though the average mesial movements of premolars (anchorage losses) increased slightly after 14 and 28 days of consolidation than those on day 1, no significant difference was found among the variables.

**Table 1:** Amount of incisors distalizations and mesial movement of the premolars.

	Teeth movement (mm)					
	Incisors			Premolars		
1-day	4.53±0.10	5.14±0.05	4.02±0.12	1.32±0.12	1.19±0.07	1.12±0.05
14-day	4.40±0.11	4.32±0.08	4.92±0.03	1.58±0.08	1.47±0.04	1.14±0.03
28-day	4.57±0.02	4.26±0.10	4.08±0.02	1.44±0.04	1.20±0.06	1.39±0.07

### Radiographic assessment

Radiographic examination showed no evidence of complications in any of the animals, including root fracture, ankylosis, and alveolar bone height resorption. However, root resorption was found from the periapical radiographs of incisors in the experimental group. There were slight lateral root surface irregularities on the compression side and slight blunting of the root apex of the third incisors (Fig. 4), whereas root resorption of other incisors was not obvious. But all the incisor periodontal ligaments increased in width after the compression in the experimental group as compared with those before the compression and in the control group. No obvious dental root resorption could be observed in the periapical radiographs of control group. Since root resorption of the third incisor was most serious among all incisors, it was focused in the following histological assessment. In addition, lateral cephaloradiographs (Fig. 5) showed anterior crossbite and slight introversion of the maxillary anterior teeth in the experimental group.

### Histological assessment

The representative microphotographs of H&E stained sections from the experimental group are shown in Figs 6, 7, 8. After 1-day consolidation, the periodontal ligament was widened and the fibroblasts and osteoblasts were accumulated on the tension side. There appeared to be thin projection of bone spicules along the direction of tooth movement. On the compression side, the periodontium narrowed and a little hyalinization area and undermining resorption could be observed. Considerable root resorption was extended into the dentin, ranging from the cementoenamel junction to the root apex. Obvious blood vessel dilatation and congestion in the pulp could also be observed.

After 14-day consolidation, the periodontal ligament was still widened and the new trabecular bone was striated. Superficial osteoid calcified partly, and well-organized osteoblasts could be seen on the tension side. The osteoclast-absorbed osseous tissue in direct and undermining resorption was still seen on the compression side. Except for significant inflammatory cell infiltration, blood vessel dilatation and congestion markedly decreased in the pulp than those on day 1. After 28-day consolidation, the periodontal ligament on both sides reverted to the normal status and new lamina dura formed. Partial repair, with the resorption cavity walls partly covered with cementum, was observed on the root surface. Using high-power microscopic lens, some cementoblasts could be seen distributed on the repaired cementum. Most of the congestion in the pulp had disappeared except for a small part of infiltrated inflammatory cells.

No change in bone remodeling was found in the H&E stained sections of the control group (data not shown).

## Discussion

Because orthodontic tooth movement is the result of bone remodeling secondary to the mechanical force and teeth usually move at the rate of approximately 1 mm per month, distalizing the anterior teeth can take up to 8 months and even longer for certain adult patients [16]. Clinical studies indicate that the duration of orthodontic treatment is one of the most-complained issues among the patients. Recently, several groups have reported that it is possible to quickly move the canines into the first premolar extraction sites based on the DO theory. In this study, a canine model is successfully established for rapid maxillary anterior teeth retraction *en masse*. We chose beagle dogs for several reasons. Beagle dogs, a mammalian omnivore, show high similarity in their teeth structure to human teeth. Specifically, the bone density of their jaw and alveolar bone is similar to that of human's and their periodontal ligament is also similar to human tissues. In addition, as model experimental dogs, beagle dogs are easy to tame, not demanding on food, resistant to diseases, and have a high tolerance to multiple anesthesia procedures. Due to their docile nature, we could apply the daily distractor advancement and check the experimental devices in their mouth without anesthesia.

In our model, all of the anterior teeth moved together for about 4.5 mm within 12 days, a rate much faster than the traditional orthodontic tooth movement. All animals tolerated the experimental procedures well without complications, such as root fracture, ankylosis, loosening of teeth, soft tissue dehiscence, alveolar bone losses or bone necrosis in the operated region. The results of this preliminary animal model study suggest that it is possible and safe to compress the maxillary bone and achieve rapid bulk adduction of maxillary anterior teeth aided by the modified alveolar corticotomy technique. The age of the beagle dogs used in the study is 24 months, equivalent to 24 years old in humans. Therefore the current results also suggest that corticotomy-assisted orthodontic treatment can be one optimal method for adult maxillary protrusion patients because of its shorter duration of treatment than those of conventional orthodontic therapies.

Classic orthodontic tooth movement can be divided into 3 periods: initial phase, lag phase, and postlag phase. Unlike the classic tooth displacement curve [17], the dental distraction response under heavy intermittent force is approximately a straight line that cannot be divided [18]. In this experiment, the tooth displacement curve of the incisors was not studied during the distraction. But after consolidation for a certain period, there was some relapse based on the observation of teeth movement. During the compression, a little bending of the screw and bar of the distractor has been observed under the heavy intermittent force. We speculate that the flexibility of the distractor may be related to teeth relapse but the exact mechanism needs further studies.

### The mechanism of rapid maxillary anterior teeth retraction *en masse* achieved by bone compression

Rapid maxillary anterior teeth retraction *en masse* achieved by bone compression in this study is different from the technique of “distraction of periodontal ligament” developed by Liou et al. [14] or “dentoalveolar distraction osteogenesis” by Kisnisci et al. [15], both of which are essentially based on the principles of DO. Despite distalization of the teeth to the extraction sites is targeted by all three techniques, an extra process of compressing the palatal bone at the same time of decortication is introduced in this study, which distinguishes the present technique from the other two. Moreover, the current maxillary anterior teeth retraction *en masse* in the form of teeth-premaxilla complex is quite different from the single tooth by Liou et al [14] and dentoalveolar distraction by Kisnisci et al [15].

In the dental distraction studies from the above two groups, heavy intermittent force is applied to the canines with screws. The bend and fracture of the interseptal bone are believed to be the main reasons for rapid canine retraction [18]. However, several studies suggest that bone formation with corticotomy-assisted tooth movement occurs by RAP, a term initially coined to describe the rare cases of fracture healing [7], [9]. Several mechanisms of RAP have been proposed, including a decrease in the osteoblast cell number, cell proliferation responses, neovascularization, and local and systemic mediators. Corticotomy-assisted tooth movement is associated with lack of hyalinization and early tartrate-resistant alkaline phosphatase staining [19]. The variability in tooth movement rate is affected by the presence of hyalinization [20], [21]. In this study, less hyalinization was observed on the compression side of the experimental group. It might be the result of RAP, which was associated with increased systemic inflammation markers [22] and a shift in the number of osteoclast and osteoblast cell populations resulting in an osteopenic effect [23]. The mechanism for different periodontal tissue changing and osteotomic healing between the experiment and control groups under compressive force is next major question. The profiles of chemokines, such as the osteoprotegerin/receptor activator of nuclear factor-κB ligand signaling system, expressed in osteoclasts and osteoblasts for osteogenesis in response to mechanical stress have been examined and will be reported in another article as a follow-up study of this canine model.

The loss of posterior teeth anchorage is less even if there is only a little relapse during the four weeks of immobilized observation, therefore achieving a high clinical level of anchorage. The traditional view believes that most of the loss of posterior teeth anchorage is caused by excessive orthodontic stress. But in the course of rapid tooth movement in the distraction of periodontal ligament and DAD, the majority of molars actually do not move to the mesial. Only a small number of teeth have minute mesial movements with almost no loss of anchorage [14]. Liou et al. believe that this can be explained by the time gap between the stress application on the canine and anchorage teeth. After the alveolar bone is partially removed and the resistance is reduced, as the canine is moved rapidly to the distal end, the anchorage teeth still remain in the lag phase of tooth movement during the second and third weeks. In this case, the loss of anchorage is minimal. Therefore, to prevent the loss of anchorage, the authors emphasize that the canine should be drawn to the intended position within three weeks. This view has been generally proved by current experimental findings.

### Root resorption

The root apices of the third incisors were not intact and clear in most of the periapical films of the experimental group. Although periapical radiographs are still an important tool available for detecting root resorption in daily clinical practice, they are not adequate enough to accurately measure resorption. Tissue slices are frequently used to evaluate root resorption. It is generally accepted that some root resorption will occur with any orthodontic tooth movement, and various conditions may affect root resorption [24]. Microscopic examination of the current slices showed some serious external root resorption of the third incisors in the experimental group, especially after 12 days of compression and 1 day of consolidation. Partial repair was observed after consolidation for 28 days. We have treated another batch of beagle dogs with 0.75 mm per day compression and found similar results of root resorption and repair (data not shown). Numerous studies have attempted to describe root resorption repair sequences [25]–[27]. According to Owman-Moll et al. [28], approximately 75% of root resorption will be repaired by the formation of cellular cementum on the resorbed surface of the dentin. This process of forming resorption lacunae followed by cementum “healing” is considered normal and does not predicate root resorption, unless the ability of cementum regeneration is less than the extent of resorption lacunae formation. In general, the duration of the applied force is a far more critical factor for the root resorption than the magnitude of the force [29]–[32]. In spite of the heavy force applied, the incisors were distalized for only 12 days in this study. It appears that the initial strain on the teeth resulted in resorption lacunae formation as previously reported, and that later cementum repair can occur as predicted. Therefore, the current findings imply the normal process of transitory rather than permanent root resorption. The long-term effects of current treatments on root resorption need further study. In addition, this study indicates that the third incisor is typically the foothold of all incisors. A three-dimensional finite element analysis is necessary to disclose the strain distribution over the compression area in order to avoid harmful stress on the third incisor.

### Pulp vitality

A modest and transient inflammatory response in the pulp is observed after routine application of orthodontic force in a previous study [33]. But in this study, the pulp response was much more evident. Blood vessel dilatation and congestion in the pulp with significant inflammatory cell infiltration was obvious one day after the compression ended. Although the symptoms ameliorated with the time, there were still a small part of infiltrated inflammatory cells after consolidation for 28 days. But no necrosis was observed. Long-term effects on pulp vitality after rapid movement have not been described in the literature. Liou et al. [14] demonstrates that the pulp is still vital in an animal experimental research even if the tooth was moved rapidly at the rate of 1.2 mm per week. Therefore, pulp vitality deserves additional investigation.

### Future directions

This study is a preliminary study on the feasibility of rapid maxillary anterior teeth retraction *en masse* with a compressive device. Before this method can be applied clinically, many issues need to be addressed by future studies. First, the extent of pulp inflammation and root resorption, two common symptoms under conventional orthodontic force, need to be closely monitored. Under conventional treatment, these two symptoms are often healed or restored when orthodontic force is stopped, suggesting that they can be tolerated by the body and are acceptable by clinicians. Although the force applied in our study is heavier than that used in traditional orthodontic treatment, we still observed restorative responses of the teeth. This phenomenon indicates that pulp inflammation and root resorption may also be acceptable in our model. To further minimize the occurrence of these two symptoms, we suggest reducing force from 1 compression/day, 0.5 mm/time, which was used in this study, to 2 compressions/day, 0.25–0.35 mm/time.

Another important issue is pain level. Although we did not analyze pain level in this study, animals did not show any abnormal behavior, suggesting that the postoperative pain was not severe. Furthermore, the daily distractor advancement was applied in the absence of anesthesia or coercive measures. Held gently in an experimenter's hand, although beagle dogs did try to avoid the advancement, they nevertheless endured the operation well. This observation suggests that the discomfort or pain induced by our procedure is within the tolerable range. This is similar to the reactions of clinical patients under distraction osteogenesis.

Furthermore, in future clinical applications, to make patients more comfortable, the metal band used in this study can be replaced with periodontal splinting, which is smaller and more aesthetically acceptable. In addition, the distraction device applied in this study can also be replaced with a smaller, more refined distraction device.

### Conclusions

Through animal experiments, this study proves that it is feasible and safe to achieve rapid maxillary anterior teeth retraction *en masse* with a compressive device and aided by modified alveolar surgical methods. Active bone deposition in the tension area and bone resorption in the compression area ensured rapid teeth movement. But root resorption and pulp inflammation were observed during the distraction. Although they partially recovered after consolidation, the long-term effects on root resorption, pulp vitality, and periodontal tissue health need further evaluation. Under this compressive force, whether the process of callus healing will change or not and what is the underlying mechanism are the next major questions we plan to solve.

This is a pilot, animal model study to achieve rapid anterior teeth movement *en masse*. If it can be applied to the clinic, it will greatly reduce the duration of orthodontic treatment. But we should also note that the compressive device is too big to make patients feel comfortable and the alveolar surgical method which involves the upper jaw may deter patients from seeking the treatment. Therefore, there is still a long way to go to apply this method to clinical use.

## Materials and Methods

### Animals

Twelve 24-month-old male beagle dogs, weighing 12–16 kg were selected in this study. They all had intact maxillary and mandibular permanent teeth showing normal occlusion and good systemic and periodontal health at the beginning of the experiments. The animals were housed in an animal facility with a controlled environment at the Animal Experiment Center of Sun Yat-sen University and fed with standard laboratory diet and free access to drinking water. The housing, care, and experimental protocols were in accordance with the guidelines of the Medical Institutional Animal Care and Use Committee of Sun Yat-sen University. After arrival at the facility, all animals were quarantined for no less than one week. The protocol was approved by the Academic and Medical Ethic Committee of Guangdong Provincial Stomatological Hospital, Southern Medical University (Permit Number: 2008025).

The first dog was used to standardize the procedures and optimize the efficiency of the interventions; the remaining 11 dogs started the experiments 4 weeks later. Before each intervention, the animals were sedated with 3% sodium pentobarbital (30 mg/kg, intramuscularly, Merck, Germany).

### Fabrication of the compressive device

The cone-shaped tooth coronals of the third incisors and premolars made it difficult to keep the experimental device in place, so coronal preparation was first carried out with a high-speed turbo drill to increase the retention on both sides of the third incisors and the second and third premolars. The light-curing composite resin (3 M, USA) was then applied to shape the coronal contour into a column. Finally, the bilateral maxillary impressions were taken with a rubber-based impression material (Heraeus Kulzer Dental, Germany) and an individual tray made of self-curing acrylic resin. Based on the cast, individual bands for the anterior teeth and premolars were fabricated. The bands of the anterior teeth as well as the second and third premolars were designed united to strengthen the anchorage.

**Figure 1:**
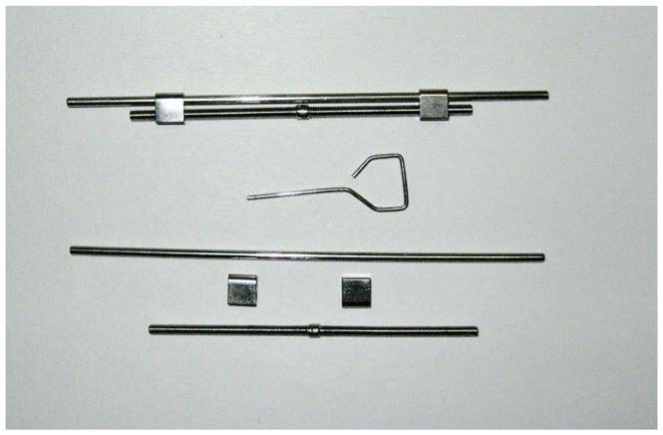
Custom-made appliance for distraction.

**Figure 2:**
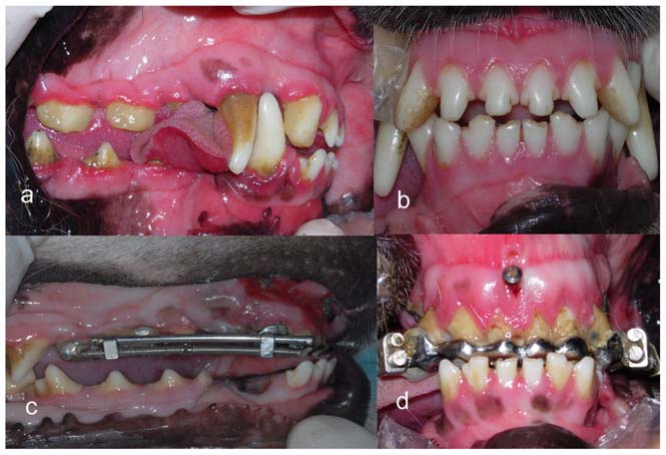
Occlusion comparison of experiment groups before and after the treatment. (a, b) before the treatment. (c, d) after the treatment. Occlusal views show that the upper incisors were retracted after 12 days of distalization.

The distractor was designed on the basis of an orthodontic expander (Xinye Dental Odontological Materials, China), which was activated using a key. A half turn (180°) activation of the screw could produce 0.5 mm of distal movement (Fig. 1). The length of the screw was designed according to the distance between the third incisor and the second premolar. The custom-made distractor was soldered on the third incisors bands against the second premolars to setup the complete tooth-borne compressive device (Fig. 2). Before cementing the compressive device, alveolar surgery was performed.

### Surgical procedures

**Figure 3:**
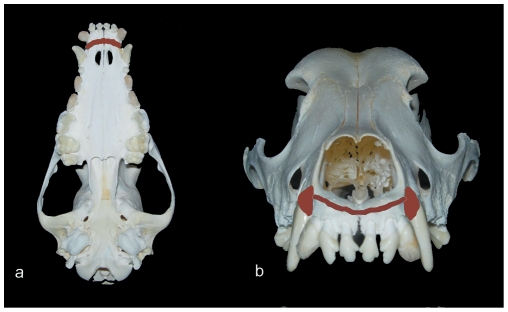
Areas of corticotomy (brown). (a) area on the palatal side. (b) area on the buccal side.

All surgical procedures were performed under general anesthesia with sodium pentobarbital and local infiltrated anesthesia with 2% lidocaine hydrochloride (Mingxing Pharmaceutical, China). Under aseptic conditions, following the maxillary canine extraction, the labial and palatal cortical plates of the extraction sockets were grinded and removed with a round carbide bur on both sides, and extended obliquely towards the lower lateral margin of the pyriform aperture to weaken its resistance.If the nasopalatal neurovascular bundle was well protected, the palatal mucoperiosteal flap was elevated from the palatal alveolar crest of the six anterior teeth to the apical region to expose the cortical bone around them. The corticotomy was performed with a round carbide bur inserted just into the marrow space under water cooling, and the palatal corticotomy cut stopped right at the extraction sockets laterally. Then a horizontal labial incision was made parallel to the gingival margin of the maxillary incisors beyond the depth of the vestibule. After the lower margin of the pyriform aperture was exposed, corticotomy was performed with a small round carbide bur along the margin to connect the bilateral extraction sockets (Fig. 3). Additionally, a titanium mini-plant as a marker was placed in the palatal middle of the distal margins of the first two maxillary molars, which was vertical to the palatal bone surface. The palate mucoperiosteal flap was repositioned and the wound was secured by interrupted 1-0 silk sutures after corticotomy. Another marker was placed in the middle of the root apex of two upper central incisiors, which was vertical to the labial surface. The tooth-borne compressive device was then cemented in place. After that, both sides of the mandibular canines were delivered in order to avoid interfering with the occlusion. All animals were daily administered penicillin G (800,000 units, intramuscularly) as antibiotic prophylaxis for one week.

**Figure 4:**
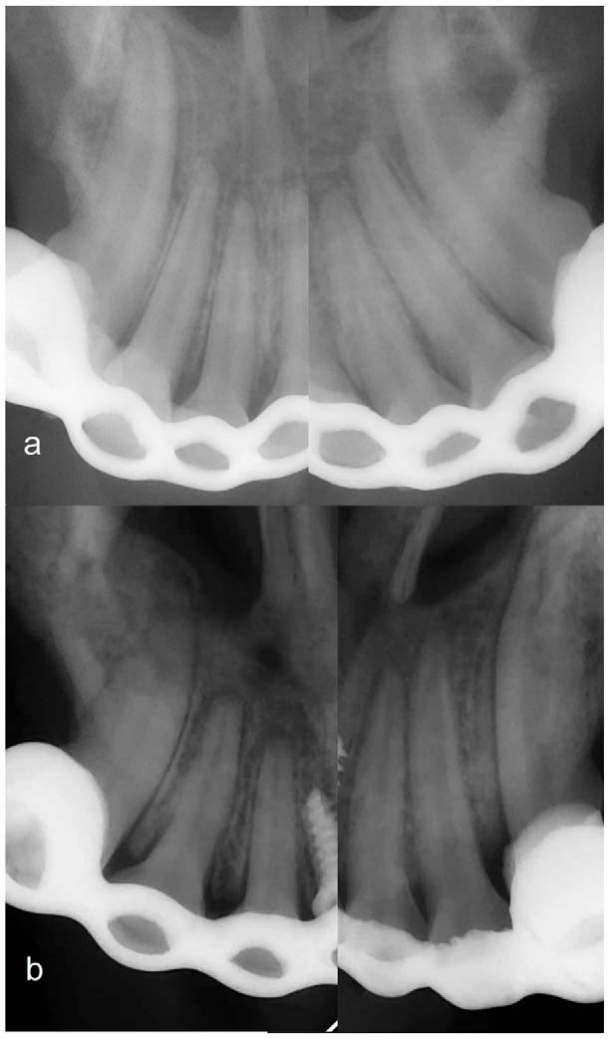
Periapical films of maxillary anterior teeth. (a) control group. (b) experiment group. The films show some root defects on the compressive side of the third incisors in the experiment group after compression for 12 d and consolidation for 1 d. All the periodontal ligament space was thicker in the experimental group than the control group.

**Figure 5:**
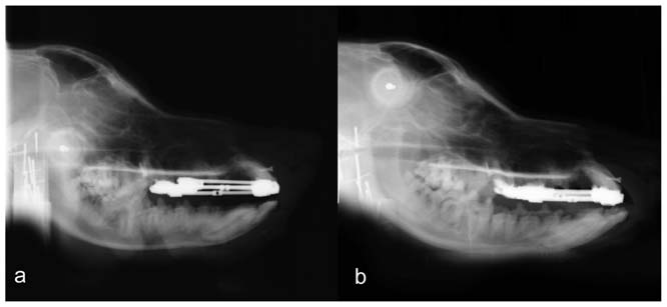
Cephalometric films of experiment group before and after treatment. (a) before the treatment. (b) after compression for 12 d and consolidation for 1 d. The films show a little introversion on incisors after 12 days of distalization.

**Figure 6:**
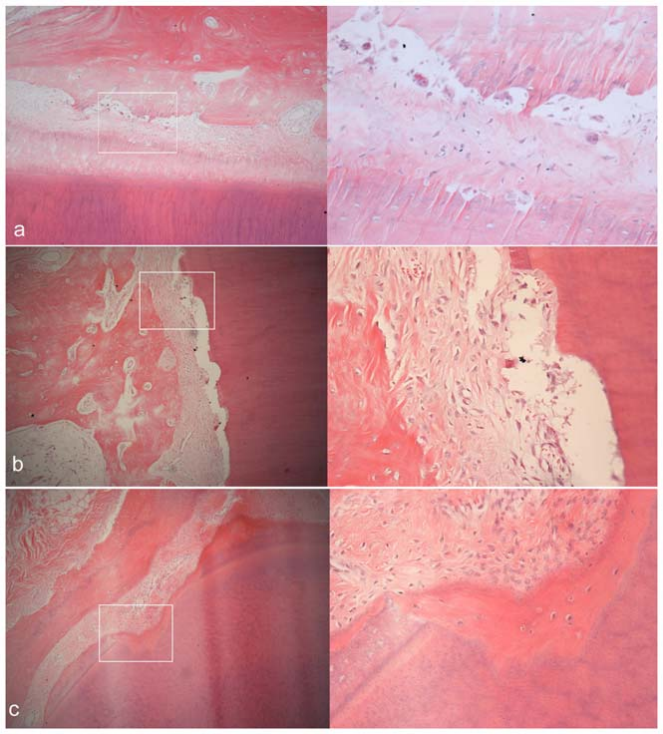
Histological microphotographs of compressive side of third incisiors in experiment groups. Each panel includes two pictures of H&E staining with the amplifications of 100× (left) and 400× (right). (a) alveolar resportion after compression for 12 d and consolidation for 1 d. (b) root defects deep into the dentine after compression for 12 d and consolidation for 1 d. (c) after 28 d of consolidation, repaired cementum covering the root defects and some cementoblasts distributed inside.

**Figure 7:**
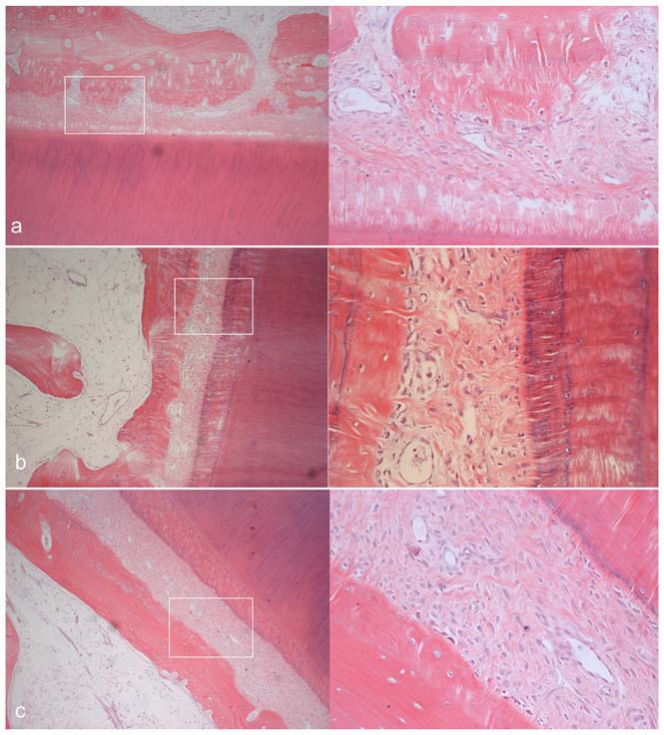
Histological microphotographs of tension side of third incisiors in experiment groups. Each panel includes two pictures of H&E staining with the amplifications of 100× (left) and 400× (right). (a) thin projection of alveolar bone spicules after compression for 12 d and consolidation for 1 d. (b) new bone formation accompanied by well-organized osteoblasts and extended, dense periodontal ligament space after 14 d of consolidation. (c) periodontal ligament space reverted to the normal status and new lamina dura formed after 28 d of consolidation.

**Figure 8:**
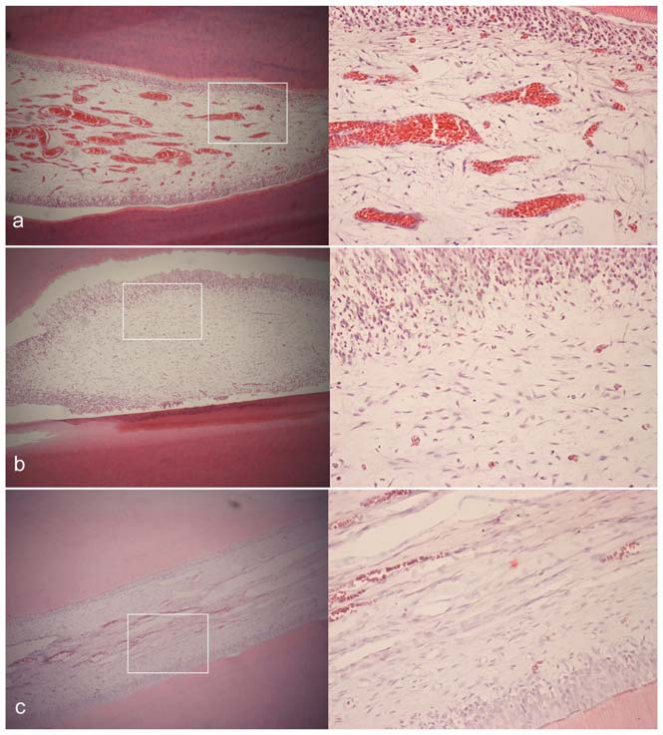
Histological microphotographs of pulp tissue of third incisiors in experiment groups. Each panel includes two pictures of H&E staining with the amplifications of 100× (left) and 400× (right). (a) obvious blood vessel dilatation and congestion in the pulp after compression for 12 d and consolidation for 1 d. (b) significant inflammatory cell infiltration in the pulp after 14 d of consolidation. (c) reverted to normal after 28 d of consolidation.

### Compression protocol

The animals were randomly assigned to the experimental or the control group. For nine dogs as the experimental group in the study, their distractors were activated immediately after the surgery because the time span was critical for the teeth movement. The distractors were activated 0.5 mm per day, continuously for 12 days to distalize the anterior teeth. After the teeth movement was completed, the distractors were left in place for consolidation. The remaining three dogs as the control group received no compression. Three animals in the experimental group were randomly selected to be euthanized by excess anesthesia after consolidation for 1 day, 14 days and 28 days, respectively. And one in the control group was sacrificed at the last day of consolidation. Each dog underwent radiographic and histological examinations after sacrifice.

### Radiographic study

The dog was placed in a custom-made box with the head fixed outside. The periapical radio videography (RVG) of maxillary incisors were taken immediately after the surgery (before the compression) and before sacrifice. The X-ray tube (Planmeca Intra, Finland) was positioned 5 cm from the animal's teeth. The machine settings were 63 kV, 8 mA with 2 s exposure time. A digital lateral cephaloradiograph was obtained with another X-ray tube (Sirona, Germany) 7 cm from the animal's right-side face and the machine settings were 73 kV, 15 mA and 9.4 s exposure time. The changes in the periodontal ligaments, alveolar bone deposition and resorption, root resorption of the maxillary teeth, as well as anterior occlusion were evaluated before and after the compression.

### Histological study

The maxillary segments, including the maxillary incisors and extraction sockets, were dissected after removing the compressive device and fixed in 10% formalin for 48 h and then decalcified with 10% EDTA for 60 days. After decalcification, the specimens were imbedded in paraffin, sectioned sagittally (6 to 8 µm) with a microtome, and stained with hematoxylin and eosin (H&E). The histological observation was performed using a conventional bright-field light microscope (Olympus CX41, Japan) and the images were captured by a camera (Olympus Q Imaging MicroPublisher 5.0 RTV, Japan). The changes in the periodontal ligaments, alveolar bone deposition and resorption, root resorption of the maxillary teeth, and pulp condition were evaluated after the compression.

### Teeth movement measurement

The distance between the reference markers on the palatal and the united points of the maxillary central incisor bands was measured to 0.02 mm with a sliding caliper (Sanfeng, Japan). So was the mesial margin of the bands on the second premolars with the palatal markers.

### Statistic analysis

The statistical analysis was performed using SPSS 13.0 program (PASW, USA) and the results were shown as mean ± standard deviation (SD).
